# Modulation of oxidative stress and P53/PCNA signaling by glucosodiene-loaded nanoferrites (GLONF) in ehrlich solid tumor–induced hepatotoxicity

**DOI:** 10.1038/s41598-026-45548-4

**Published:** 2026-04-13

**Authors:** Ehab Tousson, Afaf El Atrash, Mariam Y. Abdelrasol, Amina I. Ghoneim

**Affiliations:** 1 Zoology Department, Faculty of Science, Tanta University, Tanta, Egypt; 2 physics Department, Faculty of Science, Tanta University, Tanta, Egypt

**Keywords:** Ehrlich solid tumor, Glucosodiene Loaded over Nanoferrites, Liver, Oxidative stress, Immunohistochemistry, Biochemistry, Cancer, Drug discovery, Oncology

## Abstract

Ehrlich solid tumors are commonly used as a model to study tumor growth and evaluate potential anticancer therapies. Current study aimed to assess the curative efficacy of glucosodiene Loaded over Nanoferrites (GLONF) against Ehrlich solid tumor (EST) induced hepatotoxicity. Fifty female mice were randomly allocated into five equal groups (Control; GLONF; EST; GLONF + EST; EST+GLONF). Our findings revealed that the EST group exhibited significantly increased levels of aspartate transaminase (AST), alanine transaminase (ALT), alkaline phosphatase (ALP), hepatic injury indices, malondialdehyde (MDA), proliferating cell nuclear antigen *(*PCNA), and apoptotic p53, while showing significant depletion of albumin, total proteins, catalase, superoxide dismutase (SOD) and reduced glutathione (GSH) when compared to the control group. GLONF post-treatment (EST+ GLONF) is a highly effective strategy, demonstrating potent antitumor where it reduced tumor size, hepatoprotective, and antioxidant actions, restoring liver integrity, modulating key molecular markers and improving histological and immunohistochemical profiles.

## Introduction

Cancer is a complicated hereditary illness marked by abnormal cellular behaviours such as uncontrolled growth, invasion, and metastasis^1,2^. Breast cancer is the leading cause of cancer-related death among women worldwide, with more than three million new cases reported in 2022^3^. Breast cancer is a heterogeneous disease that caused by the uncontrolled proliferation of epithelial cells that line the breast’s ducts and lobules^4,5^. Its growth is fuelled by a mix of genetic alterations, hormone effects, and lifestyle/environmental variables^6^. Ehrlich tumors, also known as Ehrlich ascites carcinoma (EAC) or Ehrlich solid carcinoma (EST) depending on their form, are a transplantable murine tumor model originally derived from a spontaneous mammary adenocarcinoma in mice^7–9^. EST simulated breast cancer and it is a fast-growing, aggressive tumor in mice, ideal for studying cancer biology and tumor immunology due to its aggressive nature^10,11^. EST model is widely used to evaluate anticancer drug efficacy (chemotherapy, metabolic inhibitors, natural compounds) or to analyze oxidative stress, apoptosis, and cell signaling pathways^12^^13^.

Recent scientific research has increasingly focused on metabolic strategies for cancer treatment, taking advantage of the altered metabolic processes characteristic of cancer cells^14^. The glucosodiene polymer, a novel molecule derived from glucose, has demonstrated potential in inhibiting glucose metabolism and altering the acidity of the tumour microenvironment. The theory proposes treating cancer by disrupting glucose metabolism and modifying the tumor microenvironment^15^. The primary aim is to disrupt tumour metabolic activity, thereby potentially regulating disease progression^16^. In addition, glucosodiene may exert anticancer effects through multiple mechanisms, including interference with glucose metabolism, modulation of signalling pathways, and enhancement of immune responses^17^.

Glucosodiene is a new polymer made from glucose and sodium bicarbonate. It is designed to block cancer cells’ abnormal use of glucose (the Warburg effect) and reduce tumour acidity by limiting lactic acid production or buffering the tumour environment^18,19^. This dual action offers a novel treatment class that combines metabolic disruption with microenvironment remodelling. A novel Sr₀.₅Mn₀.₅La₀.₀₂Fe₁.₉₈O₄ spinel nano-ferrite was synthesized and incorporated into a tri-component nano-platform with chitosan and glucosodiene. While magnetic ferrites, biopolymer coatings, and metabolic anticancer agents have been studied individually, their integration into a single multifunctional system with validated biological performance remains insufficiently explored. This study addresses that gap by developing a synergistic nano-system that combines magnetic targeting, biocompatible drug delivery, and metabolic tumor disruption. The work further evaluates both therapeutic efficacy and biological safety, providing a more comprehensive approach to cancer nanomedicine.

A nanotechnology-based drug delivery system might offer a potential solution to the above issues^20,21^. This sort of technology may comprise a formulation or device that permits a therapeutic molecule to selectively reach a location of interest and act without harming non-target cells, organs, or tissues^22,23^. One of the most investigated, promising, and simple methods for transporting therapeutic chemicals in the body is to use nano entities as delivery vehicles^24,25^. Thus, current study sought to estimate the therapeutic efficiency of glucosodiene-loaded nanoferrites (GLONF) in EST -induced liver toxicity, oxidative stress, apoptosis, and cellular proliferation in female mice.

## Materials and methods

### Synthesis of nano-spinel ferrites via citrate sol-gel auto-combustion

Nanocrystalline Sr_0.5_Mn_0.5_La_0.02_Fe_1.98_O_4_Spinel nano-powders were produced by Citrate-based Sol-Gel Self-Combustion regimen according to the following routine^26^.

0.5 Sr(NO_3_)_2_ + 0.5 Mn(NO_3_)_2_.4H_2_O + 0.02 La(NO_3_)_3_.6H_2_O + 1.98 Fe(NO_3_)_3_.9H2O + 8 NH_4_OH + 3 C_6_H_8_O_7_ + 6 O_2 → _Sr_0.5_Mn_0.5_La_0.02_Fe_1.98_O_4_ + 8 NO_3_ + 8 NH_3_ +39.94 H_2_O + 18 CO_2_.

Stoichiometric amounts of Metal nitrates (Sr(NO_3_)_2_, Mn(NO_3_)_2_.4H_2_O, La(NO_3_)_3_.6H_2_O and Fe(NO_3_)_3_.9H2O) are dissolved in distilled water and preserved at 10 °C for 1 h. The reacting-agents are constantly stirred using a magnetic stirrer. Thereafter, the citric acid was added as a fuel to the metal nitrate solution in mole ratio of (citric acid: metal nitrates = 1:1) and magnetically stirred for 2 h. The blended solution’s pH was continuously checked and monitored as NH_4_OH liquid was supplied by dropping til pH ⁓ 11. By gradually raising the hot plate’s temperature (T) to 90 °C for two hours while stirring constantly, the resulting sol was transformed into a viscous gel stage. The fluid turned into an extremely thick dark gel as it evaporated. After that, the temperature of the viscous brown gel was raised until the mixture burned thoroughly, producing the Sr_0.5_Mn_0.5_La_0.02_Fe_1.98_O_4_nano-powders. To extract the fine nano-powders, fine spinel nanocrystals were then finely pulverized in a sterilizing agate mortar^26^.

### Creation of nano-spinel ferrites with chitosan (MNPs-CH)

A total of 0.5 g of **Sr**_**0.5**_**Mn**_**0.5**_**La**_**0.02**_**Fe**_**1.98**_**O**_**4**_Spinel ferrite nanoparticles (MNPs) was added to a 0.5% (W/V) Chitosan (CH) solution at pH 5 which priory was dissolved in 2% acetic acid (2% V/V) in 250 sterilizing glass beaker. Thence, the mix was sonicated at 60 °C for 1 h using Ultrasonic bath followed by stirring magnetically for 18-hours at room T. External magnetic field was used to separate the resulting black homogenous mixture (MNPs-CH), which was spread over a sterilized glass circular flat dish and then allowed to dry overnight at room T^27^.

### Synthesis of glucosodiene (GS)

Dextrose monohydrate (C_6_H_14_O_7_) and sodium bicarbonate (NaHCO_3_) were the ingredients utilized in this inspection. 2.5 g of sodium bicarbonate and 3.5 g of dextrose were dissolved in 100 milliliters of distilled water to perform a substitution reaction in order to get the final polymer molecule. After heating the mixture to 100 degrees Celsius for five minutes, bubbles appeared, confirming that CO_2_gas had been released and that the substitution reaction had taken place. The resultant product, 1–2-O-β-D-Glucopyranosyl- α-D-glucose (GS named Glucosodiene), which was dried via lyophilization device (Alpha 1–2 LDplus Lyophilizer, max pressure 25 bar, max T 120 °C, 230 V, 50 Hz, 3.5 A) and posterior to dissolving in DMSO it was subjected to NMR analysis (utilizing the JEOL JNM-ECZR 500 MHz NMR Spectrometer)^16^.

### Construction of Nano-Ferrite/Chitosan/Glucosodiene (NF/CH/GS) nanocomposite

50 mg of Glucosodiene (GS) was dissolved in 50 ml distilled water at 100 °C under stirring for 8 h, thereafter; the solution was left to cool at room T. Then, 100 mg of Sr_0.5_Mn_0.5_La_0.02_Fe_1.98_O_4_ Spinel ferrite/Chitosan nanocomposites (MNPs-CH) was dispersed in 50 ml distilled water and sonicated for 2 h, then 50 µL of Glutaraldehyde was added to this solution and then all was magnetically stirred for 18 h. Thence, GS solution was added by dropping to MNPs-CH solution with the ratio 1 : 2, respectively; which was subjected to sonication for 2 h at room temperature, then magnetic stirring for 18 h at room T. Thence, the final obtained product was freeze-dried and Sr_0.5_Mn_0.5_La_0.02_Fe_1.98_O_4_/Chitosan**/**Glucosodiene (NF/CH/GS) nanocomposite was obtained.

## Characterization of nanoparticles

### X-ray diffraction (XRD) measurements

Nanoparticles in the form of polycrystalline Nano-powder are characterized at room temperature by XRD patterns via GNR APD 2000 Pro X-ray diffractometer step scan type and CuKα1 radiation with wavelength λ = 1.540598 Å using sample holder with active sample window of 20 mm diameter and depth of 1–2 mm.

Lattice parameter *a* for Nano-spinel crystals was elicited utilizing the expression;

*a* = $d_{{hkl}}^{{}}$(h^2^ + k^2^+l^2^)^1/2^, where (hkl) are miller indices and *d*_hkl_ is the inter-planer distance obtained by Bragg’s expression: n λ = 2 $d_{{hkl}}^{{}}$ sin θ reflection order *n* = 1, θ is Bragg’s angle.

The Lattice parameter a = 8.4 Å which agrees well with its standard values.

The crystallite size R was determined using the higher intensity diffraction peak (311) and Sherrer’s formula:$R=\frac{{0.9\lambda }}{{{\beta _{\frac{1}{2}}}\cos \theta }}$ where ${\beta _{\frac{1}{2}}}$ is the full width at half maximum of the peak (311).

### Transmission electron microscopy (TEM) Imaging and VSM measurements

High Resolution TEM with Acceleration Voltage 200 KV **(**HTEM; JeoL-JEM- 2100 PLUS, Japan), where the sample powder was first dispersed in pure ethanol by ultrasonic waves for 60 min to perfectly separate the nanoparticles from each other and then the suspension was dropped on a copper grid with a carbon film.

Hysteresis loops of the samples were recorded at room temperature using the vibrating sample magnetometer, Lakeshore – 7410 and a maximum applied field upto 20,000 Gauss.

### NMR measurements

The^1^ H NMR and^13^ C NMR spectra analysis were depicted for the Glucosodiene Polymer compound, 1–2-O-β-D-Glucopyranosyl- α-D-glucose after dissolving it in DMSO utilizing the JEOL JNM-ECZR 500 MHz NMR Spectrometer.

### Animals

Fifty female CD1 mice were maintained under controlled conditions, and all experimental procedures were ethically approved and conducted according to our faculty (IACUC-SCI-TU-0458), NIH and ARRIVE guidelines.

### Induction of Ehrlich solid carcinoma (EST)

Mice with EAC were supplied by the Egyptian National Cancer Institute, and tumors were established via subcutaneous injection of 2.5–3 × 10⁶ EAC cells into the left thigh^7^..

### Experimental groups

The mice were allowed to acclimate for two weeks. 50 female mice were randomly allocated to five groups (*n* = 10 per group):

**Group 1** (Control): Negative control, mice were left untreated.

**Group 2** (GLONF): GLONF (50.4 mg/kg/day) were given to mice orally for 10 days.

**Group 3** (EST): Ehrlich solid carcinoma (EST) group mice were infused subcutaneously with 2.5 × 10^6^ cells/mouse diluted in normal saline.

**Group 4** (GLONF + EST): Mice were subcutaneously infused with 2.5 × 10^6^ cells/mouse to initiate tumor and at the same time treated with GLONF extract (50.4 mg/kg/day) orally for 10 days.

**Group 5** (EST+ GLONF): Mice were subcutaneously infused with 2.5 × 10^6^ cells/mouse to initiate tumor and left for 10 days (until the appearance of the solid tumor) then treated with GLONF extract (50.4 mg/kg/day) orally for another 10 days.

### Blood and serum samples

After completion of the treatments, the animals were anesthetized with isoflurane and euthanized by cervical dislocation. Blood samples were aseptically collected via venipuncture into plain glass tubes without additives and left to clot naturally. The samples were subsequently centrifuged at 4000 rpm for 5 min, after which the serum was separated and stored at − 18 °C until blood parameter analysis. The animals were dissected and the liver was immediately excised and divided into two portions. Small liver sections were fixed in 10% neutral buffered formalin for histopathological and immuohistochemical analyses and the residual liver tissue were assessed for subsequent biochemical estimations.

### Evaluation of serum liver functions tests

In line with Reitman and Frankel^28^, the serums aspartate transaminase (AST) and alanine transaminase (ALT) were assessed. Estimation of alkaline phosphatase (ALP) activity in sera was assessed according to El-Aarag et al.^29^ while albumin and total proteins levels were measured in serum according to Doumas et al.^30^

### Estimation of the lipid peroxidation and antioxidant assays

Portions of the liver were homogenized (10% w/v) in ice-cold sodium–potassium phosphate buffer (pH 7.4) and subsequently centrifuged at 10,000 × g for 20 min. The resulting clear supernatants were calm and kept at − 20 °C until further analysis. Lipid peroxidation was evaluated by quantifying malondialdehyde (MDA) levels according to the method described by Lahouel et al.^31^, while reduced glutathione (GSH) levels were determined following the procedure outlined by Beutler^32^..

Antioxidant capacity was assessed by measuring the activities of key enzymes, including catalase (CAT) and superoxide dismutase (SOD). These enzymatic activities were analyzed using commercially available kits in accordance with the methods reported by Saggu et al.^33^ and Yilmaz et al.^34^, respectively.

### Histopathological investigation

Livers were quickly removed, cut into tiny were fixed in 10% buffered formalin for 24–48 h, processed, sectioned at 5 μm, and stained with H&E according to the method of Bancroft and Cook^35^..

### Immuno-histochemical analysis

PCNA and P53 immunoreactivities in liver tissues were assessed using the avidin–biotin–peroxidase (ABC) immunohistochemical method on 5 μm deparaffinized sections. After blocking endogenous peroxidase activity and nonspecific binding, sections were incubated with a monoclonal anti-PCNA and anti-P53 as primary antibody, followed by streptavidin–peroxidase as described by Tousson et al.^36^ and Tousson et al.^37^ respectively. Immunostaining was visualized using diaminobenzidine (DAB), counterstained with hematoxylin, and the sections were dehydrated, cleared, and mounted for microscopic examination. The stained cells were extracted by color thresholding using Image J program to extract the brown color for quantitation.

### Statistical analysis

Data were analyzed using one-way analysis of variance (ANOVA) to determine significant differences among groups. Results are presented as mean ± standard error (SE). Statistical significance was indicated by (*) and (^#^), representing significant differences compared with the EST group and the control group, respectively, at *p* < 0.01. An unpaired t-test was used for pairwise comparisons between groups. A p-value of less than 0.01 was considered statistically significant. All statistical analyses were performed using SPSS software version 21 (SPSS^®^ Inc., USA).

## Results

### X-ray diffraction Spectra

**Fig. 1:**
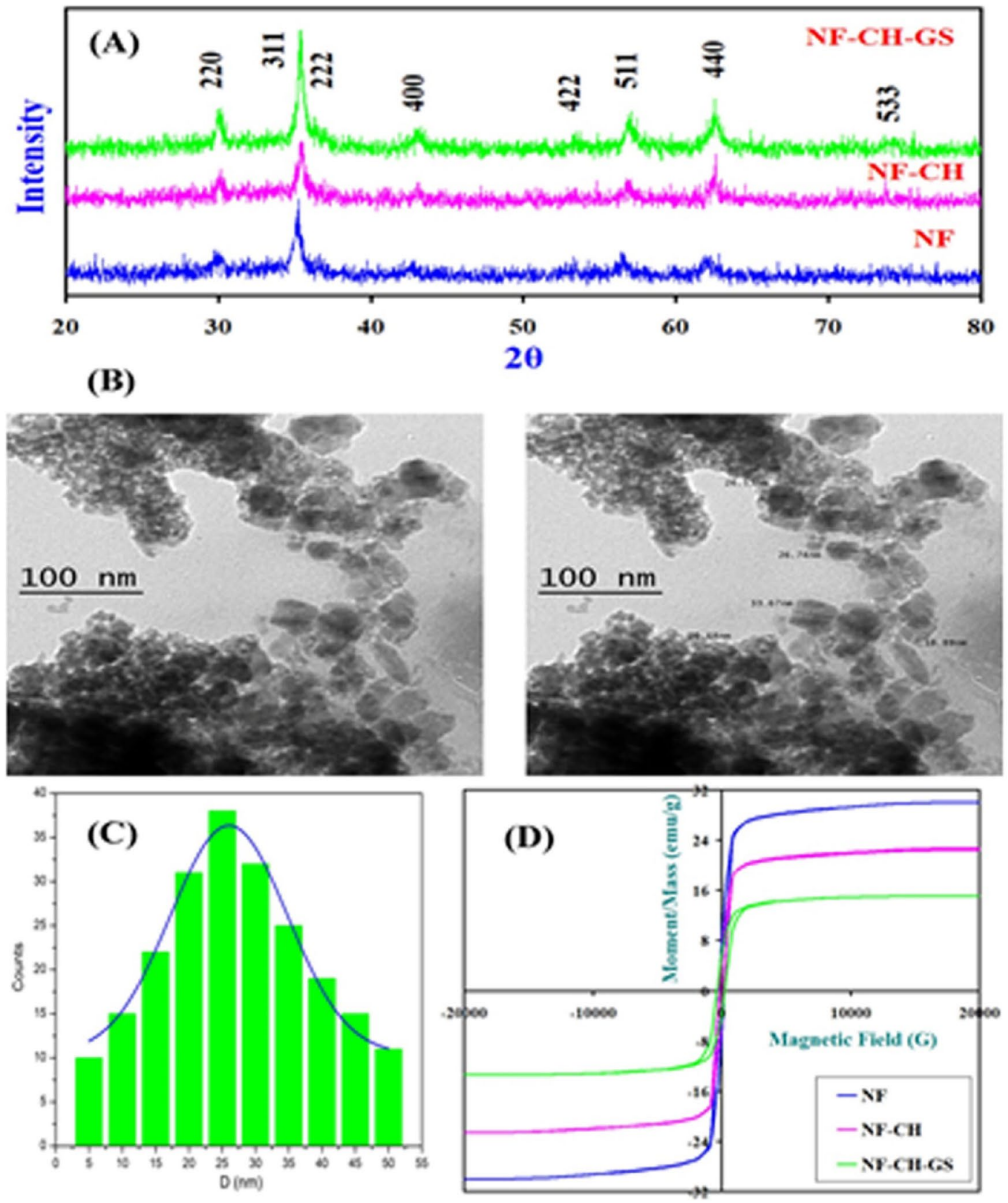
**(A)** XRD patterns for Sr_0.5_Mn_0.5_La_0.02_Fe_1.98_O_4_ Nano-ferrites (NF), NF-CH, NF-CH-GS. **(B)** HTEM image for NF-CH-GS. **(C)** Nanoparticle size distribution. **(D)** Magnetic hysteresis loops for Sr_0.5_Mn_0.5_La_0.02_Fe_1.98_O_4_ Nano-ferrites, NF-CH, NF-CH-GS.

Figure 1A illustrates the X-ray diffraction (XRD) layouts for the nanocrystalline Sr_0.5_Mn_0.5_La_0.02_Fe_1.98_O_4_ Spinel ferrite nanoparticles. XRD patterns afirmed that the nano-powders possess mono-phase Spinel nanoferrites. XRD patterns of Sr_0.5_Mn_0.5_La_0.02_Fe_1.98_O_4_ nanoparticles coated with Chitosan (NF-CH) highlighted some widening in the diffraction supremes assigning to the amorphous habit of Chitosan (CH) and indicating that Spinel ferrite nanoparticles is coated well by CH matrix. Figure 1A highlighted that CH and GS made no phase ulteration of Sr_0.5_Mn_0.5_La_0.02_Fe_1.98_O_4_ nanostructures, only makes some broadening in X-ray peaks. The development of the NF-CH-GS nano-system and the use of a CH-based cross-linked network with Sr_0.5_Mn_0.5_La_0.02_Fe_1.98_O_4_ caused the emerging supremes to broaden.

The calculated Crystallite size is ⁓ 10.8, 11.12 and 15.8 nm for as prepared Sr_0.5_Mn_0.5_La_0.02_Fe_1.98_O_4_ NF-CH and NF-CH-GS nano-system, respectively.

### Transmission electron microscope (TEM) images

Sr_0.5_Mn_0.5_La_0.02_Fe_1.98_O_4_ Spinel ferrite/Chitosan/Glucosodiene (NF-CH-GS) nano-system was examined via TEM images to ascertain their nanoparticle size, surface features, and morphological merits, and their Nanoparticle size histogram as illustrated in Fig. 1B & C. The inspected TEM pics for the NF-CH-GS nano-system viewed that the nanoparticles are aggregated with each other. Aggregation of these nanoparticles indicate the formation of ferromagnetically ordered nano-clusters. NF-CH-GS nano-system had a smooth surface, were likely spherical, and their average nanoparticle size got from HTEM was ~ 25 nm; (see Fig. 1B and C).

### Vibrating sample magnetism (VSM)

RT magnetized hysteresis loops for Sr_0.5_Mn_0.5_La_0.02_Fe_1.98_O_4_ Spinels (Fig. 1D) indicated a little hysteretic habit (low coercivity Hc) which is typical of the soft magnetic Nano-Spinels, with surging saturate magnetized field Ms = 31.12 emu/g, declining coercivity Hc is 218.33 Gauss, low remain magnetized field Mr = 5.22 emu/g and minimal squareness Mr/Ms = 0.167738.

Free Sr_0.5_Mn_0.5_La_0.02_Fe_1.98_O_4_ owed a surging saturated magnetize field (MS), which was diminished with Chitosan (CH) coating to 23.82 emu/g. Sr_0.5_Mn_0.5_La_0.02_Fe_1.98_O_4_/CH disclosed a crusial shielding impacted by CH. Hc declined upon coating with CH reaching 192.85 Gauss. Nano-powders with declining magnetic anisotropy typically showcase declining coactivity i.e. they are easy to demagnetize. Hc diminished upon CH coating, conferring a softening impact on Sr_0.5_Mn_0.5_La_0.02_Fe_1.98_O_4_. Furthermore, major shift was disclosed in magnetic features (Ms = 18.08 emu/g and Hc = 318.12 Gauss) for Sr_0.5_Mn_0.5_La_0.02_Fe_1.98_O_4_/CH/GS Nano-system, which may be assigned to the lessened surface imperfect on Sr_0.5_Mn_0.5_La_0.02_Fe_1.98_O_4_ and a dilution effect of Glucosodiene (GS).

#### FTIR Spectra

**Fig. 2:**
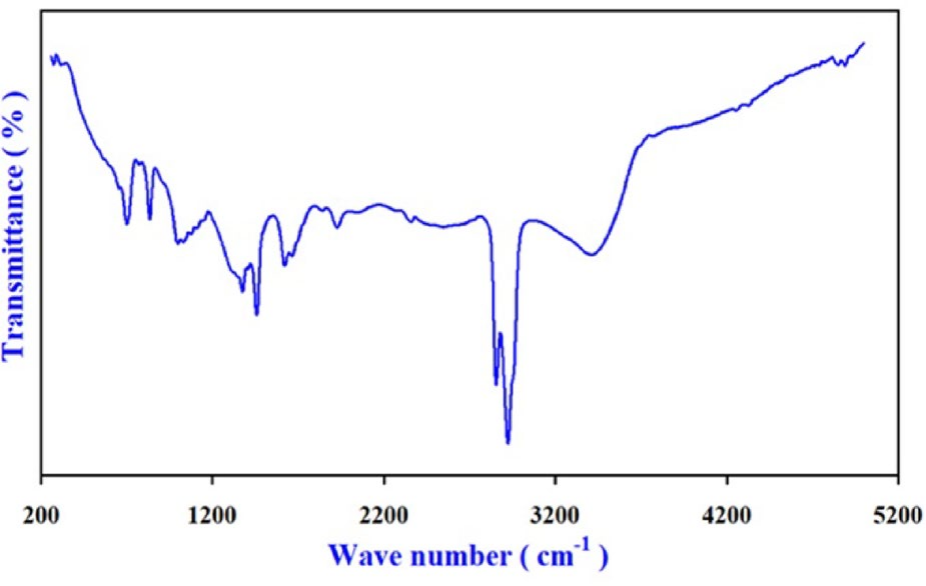
FTIR Spectrum for Glucosodiene.

FTIR spectroscopy was precisely employed to examine the molecular structure by assessing the characteristic bond energies. Figure 2 illuminated the FTIR spectra Glucosodiene (GS) reveals substantial structural modification relative to the parent carbohydrate (Dextrose (D-Glucose) while preserving a hydroxyl-rich framework.

### FTIR analysis of glucosodiene

The FTIR spectrum of glucosodiene (Fig. 2) confirms its polymeric carbohydrate structure. Low-frequency bands at 306.8 and 268.1 cm⁻¹ are attributed to skeletal lattice vibrations. Characteristic absorptions at 698.2–1651.1 cm⁻¹ correspond to C–O, C–C, C–H, and O–C–H vibrations, indicating preservation and modification of saccharide functional groups. Weak bands at 1822.8–2040.6 cm⁻¹ suggest structural reorganization within the polymeric network, while peaks at 2252.8 and 2341.5 cm⁻¹ are assigned to asymmetric CO stretching.

Strong aliphatic C–H stretching bands at 2922.1 and 2852.6 cm⁻¹ confirm retention of saturated carbon segments. A broad intense band at 3394.8 cm⁻¹, along with a sharper peak at 3757.3 cm⁻¹, corresponds to O–H stretching vibrations, indicating extensive hydrogen bonding. High-wavenumber features (4239.5–4918.3 cm⁻¹) represent overtone and combination bands of O–H stretching modes.

#### NMR Spectra

**Fig. 3:**
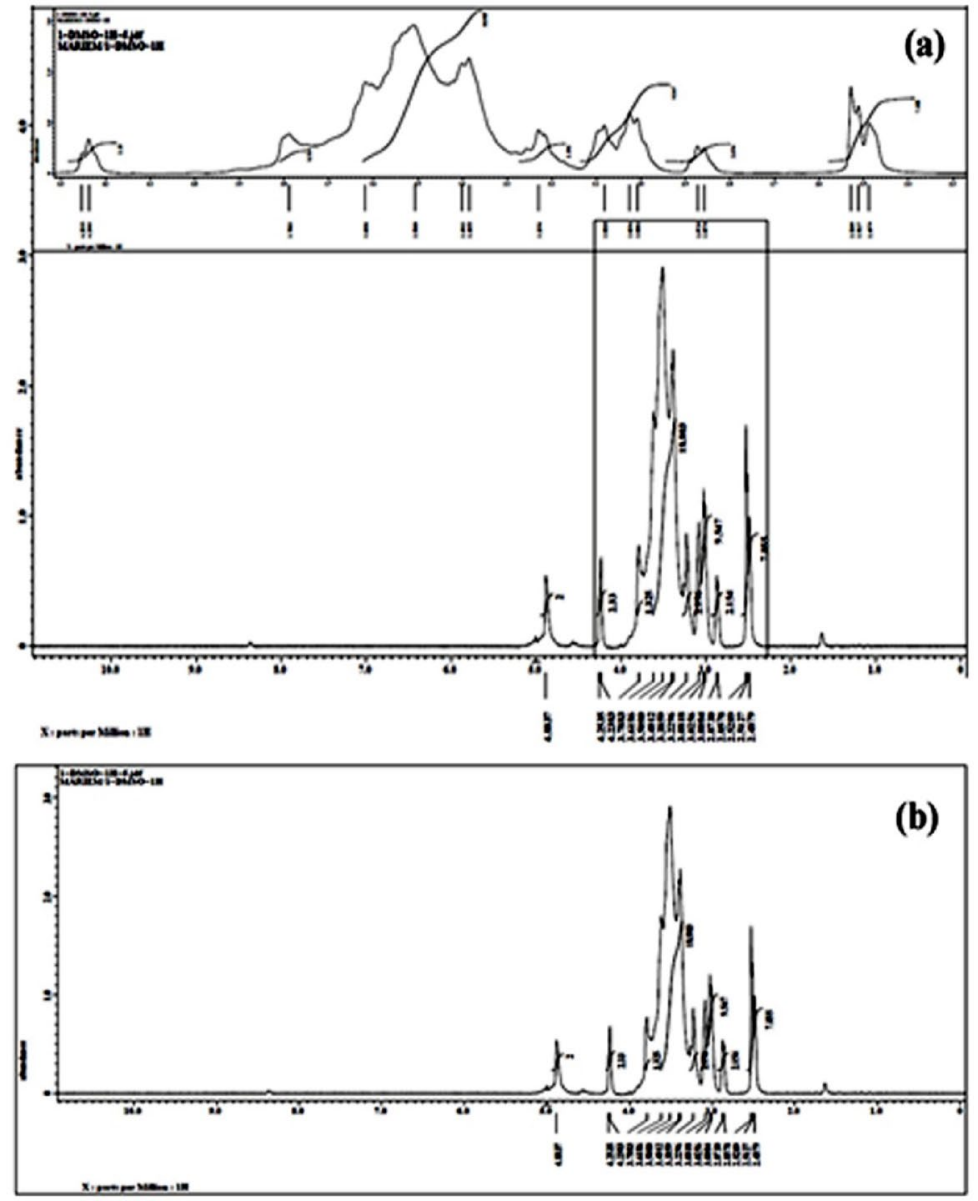
NMR-1 H spectra for glucosodiene dissolved in DMSO.

**Fig. 4:**
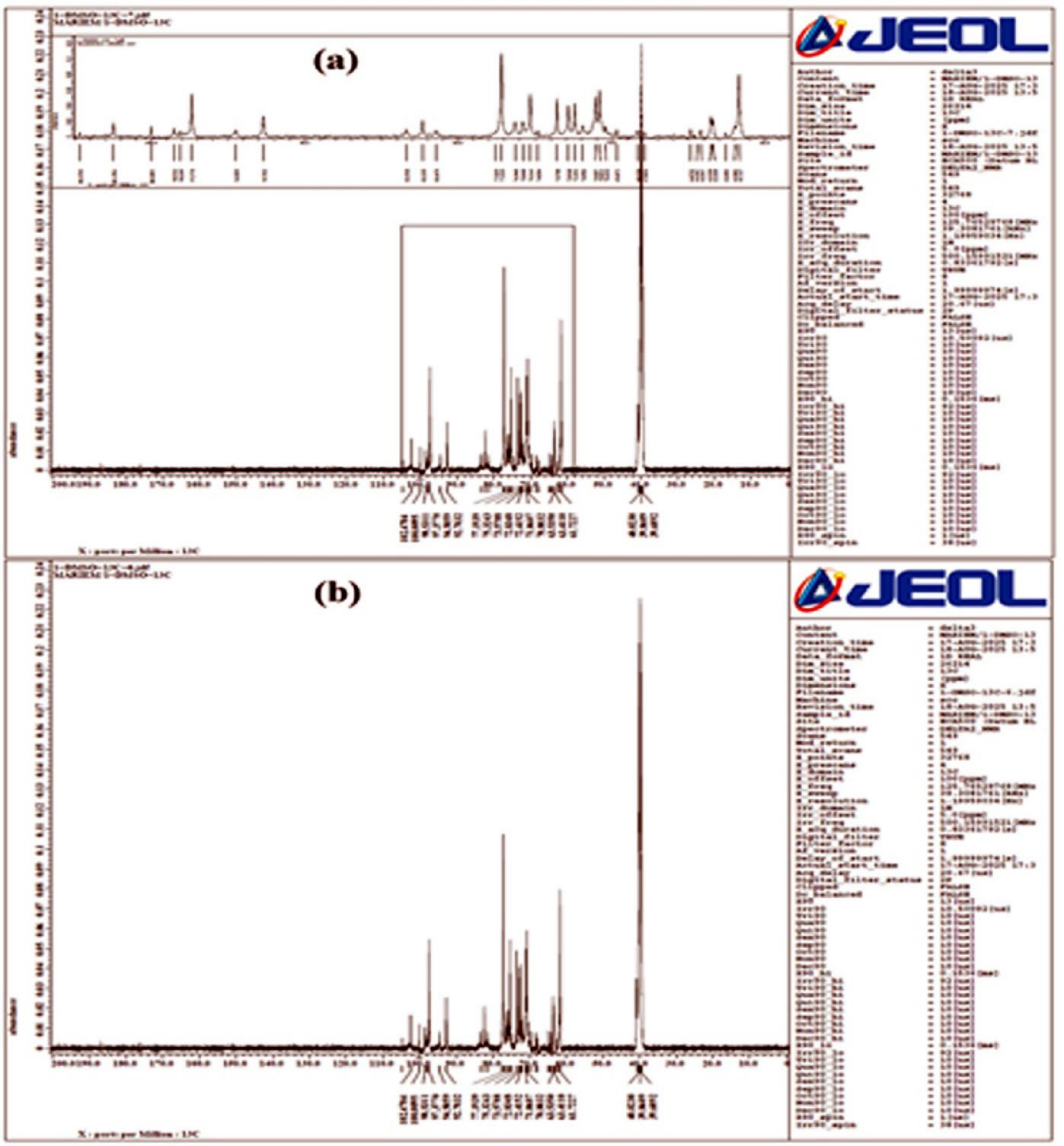
NMR-13 C spectra for glucosodiene dissolved in DMSO.

Figures 3 and 4 depicted NMR analysis (1 H NMR and 13 C NMR) of the created glucosodiene with the presented structure C_12_H_22_O_11_. For the absence of the ketone or aldehyde grouped, thence; the protruded molecule can be identified as 1–2-O-β-D-Glucopyranosyl-α-D-glucose (i.e. Glucosodiene).

The two NMR spectra of Glucosodiene (Figs. 3 and 4) provide comprehensive insight into its structure, confirming it as a glucose-derived polymer with specific modifications. The^13^**C NMR spectrum** shows multiple peaks between ~ 40–105 ppm, indicating different carbon environments: the anomeric carbons (C1) appear around 100–102 ppm, confirming glycosidic linkage formation; ring carbons (C2–C5) resonate at ~ 70–77 ppm, reflecting secondary alcohol groups; the primary alcohol carbon (C6, CH2OH) appears near 61–63 ppm; minor peaks around 40 ppm suggest carbons involved in sodium bicarbonate-mediated modifications. The^1^**H NMR spectrum** exhibits peaks from ~ 2.5–5 ppm, characteristic of sugar protons: the anomeric proton (H1) appears at ~ 4.88 ppm as a doublet (J ~ 7–8 Hz), confirming the β-glycosidic 1→2 linkage; ring protons H2–H5 resonate between 3.2 and 4.25 ppm as multiplets due to intra-ring coupling; CH2OH protons (H6) are found around 3.0–3.08 ppm; additional signals at 2.5–2.9 ppm indicate protons from modified groups introduced during synthesis. Integration of peaks aligns with expected hydrogen counts for Glucosodiene. Together, these spectra validate the polymeric glucose backbone, the presence of O-glycosidic linkages, intact ring structures, and minor chemical modifications, likely from sodium bicarbonate reactions, confirming the compound’s identity and structural integrity. The multiplet patterns and chemical shifts in both spectra provide a detailed map of hydrogen and carbon environments, illustrating the polymeric and modified nature of Glucosodiene. Peaks in^13^C NMR indicate the carbon framework, while^1^ H NMR peaks elucidate proton positions and couplings. Minor peaks in both spectra suggest selective modifications at specific carbons, highlighting chemical diversity. The anomeric signals are clear indicators of glycosidic bonding, confirming polymer formation. Ring proton multiplets reflect preserved cyclic structures and stereochemistry. CH2OH resonances corroborate primary alcohol positions. Low-field shifts (~ 2.5–3 ppm) indicate newly formed moieties or substitutions. Collectively, these data confirm Glucosodiene as a glucose derivative with both structural fidelity and functional modifications. Carbon and proton NMR assignments are consistent with literature for similar glucose polymers. The spectra’s complexity reflects polymeric heterogeneity and partial chemical modifications. Coupling constants in ^1^H NMR support β-linkage confirmation. The overall chemical shift ranges match expectations for polysaccharide derivatives. Minor deviations or extra peaks correspond to chemical modifications or derivatization effects. The interpretation validates both structural and functional aspects of Glucosodiene. It highlights the polymer’s potential reactive sites. NMR evidence aligns with synthetic methodology involving sodium bicarbonate. The spectra collectively serve as a fingerprint for compound verification, confirming glycosidic linkages, ring integrity, and modifications. Detailed peak assignments provide a framework for further chemical or biological studies. These spectra are critical for structural elucidation, quality control, and subsequent functional evaluations. Overall, the NMR data robustly support Glucosodiene’s polymeric glucose structure with specific chemical modifications, ensuring reproducibility and accurate characterization.

#### Changes in tumor weight

**Fig. 5:**
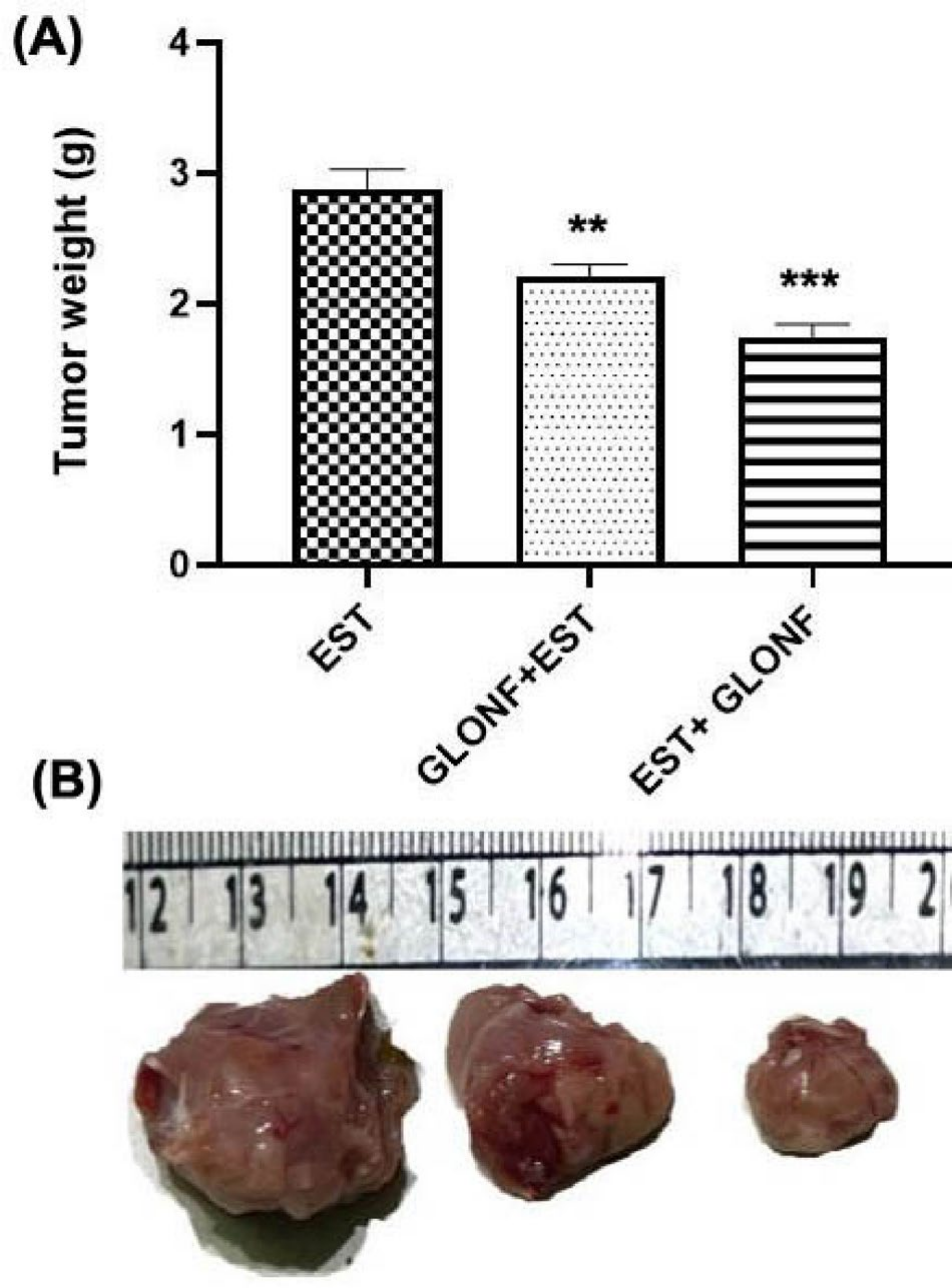
shows that treatment with GLONF (either GLONF + EST or EST+GLONF) significantly reduced tumor weight and size in mice with implanted solid tumors compared to the EST group (*p* < 0.01). The greatest reduction was observed in the EST+GLONF group, which showed a higher decrease than the GLONF + EST group.

Figure 5: Influence of GLONF on the development of tumor weight and size. (A): Histogram of tumor weight in different experimental groups. The significant difference was analyzed by unpaired *T*-test. Values are expressed as mean ± SE. The *T*-test was significant at *P* < 0 05. The unpaired *T*-test was significant from corresponding Ehrlich at NS*P* = 0 1234, ∗*P* = 0 0332, ∗∗*P* = 0 0021, ∗∗∗*P* = 0 0002, and ∗∗∗∗*P* < 0 0001. (B): Images of tumor tissues excised from different experimental groups.

#### GLONF modulated EST induced alteration in liver functions

Table 1 illustrates the changes in liver function parameters among the different experimental groups. The EST-treated group showed a significant increase (*p* < 0.01) in AST, ALT, and ALP levels, along with a significant decrease (*p* < 0.01) in albumin and total protein levels compared with the control group. In contrast, treatment with GLONF (GLONF + EST or EST+GLONF) significantly reduced (*p* < 0.01) AST, ALT, and ALP levels and significantly increased (*p* < 0.01) albumin and total protein levels relative to the EST group, with the EST+GLONF post-treatment group showing the most pronounced improvement.

#### Impact of GLONF in liver lipid peroxidation and antioxidant markers

The data presented in Table 2 demonstrate variations in lipid peroxidation and antioxidant parameters in liver homogenates across the different experimental groups. Compared with the control group, the EST-treated group exhibited a significant increase (*p* < 0.01) in malondialdehyde (MDA) levels, along with a significant decrease (*p* < 0.01) in reduced glutathione (GSH), superoxide dismutase (SOD), and catalase (CAT) activities. In contrast, treatment with GLONF (GLONF + EST or EST+GLONF) resulted in a significant reduction (*p* < 0.01) in MDA levels and a significant elevation (*p* < 0.01) in GSH, SOD, and CAT activities compared with the EST group, with the EST+GLONF group showing the most pronounced improvement.

**Table 1:** Impact of GLONF in liver functions.

Control	ALT (U/L)	AST (U/L)	Alb (mg/dl)	ALP (U/L)	T. protein (g/dl)
	33.1^#^±1.264	153.5^#^±3.753	4.28^#^±0.0286	143.1^#^±5.966	5.81^#^±0.0317
**GLONF**	28.9^#^±1.42	142.3^#^±8.066	4.19^#^±0.0735	133.3^#^±3.992	5.97^#^±0.046
**EST**	47.0*±1.472	306.4*±6.801	2.79*±0.105	234.9*±5.44	5.21*±0.053
**GLONF + EST**	41.5*^#^±1.37	222.3*^#^±9.437	3.65*^#^±0.115	192.6*^#^±4.18	5.70*^#^±0.033
**EST+GLONF**	34.8^#^±0.966	199.0*^#^±6.325	4.27^#^±0.0361	144.5^#^±3.942	6.09^#^±0.068

The significance of difference was analyzed by one – way ANOVA using computer program. Values are expressed as means ± SEM. ^#^ and * significant difference from EST and control group respectively at *P* < 0.01.

**Table 2:** Impact of GLONF in liver lipid peroxidation and antioxidant markers.

Control	MDA (nmol/g tissue)	GSH (mmol/g tissue)	SOD (U/g tissue)	CAT (U/g tissue)
	71.77^#^±1.93	2.44^#^±0.054	109.9^#^±0.86	93.8^#^±0.644
**GLONF**	74.2^#^±2.23	2.61^#^±0.0.64	115.0^#^±2.98	96.3^#^±1.037
**EST**	166.8*±1.92	0.67*±0.010	58.7*±1.868	60.97*±1.33
**GLONF + EST**	122.5*^#^±2.75	1.037*^#^±0.032	73.6*^#^±2.75	70.67*^#^±2.728
**EST+GLONF**	79.07^#^±1.44	2.07^#^±0.044	97.7*^#^±2.45	89.4^#^±3.23

The significance of difference was analyzed by one – way ANOVA using computer program. Values are expressed as means ± SEM. * and^#^ significant difference from control and EST group respectively at *P* < 0.01.

### Impact of GLONF in liver structure

**Fig. 6:**
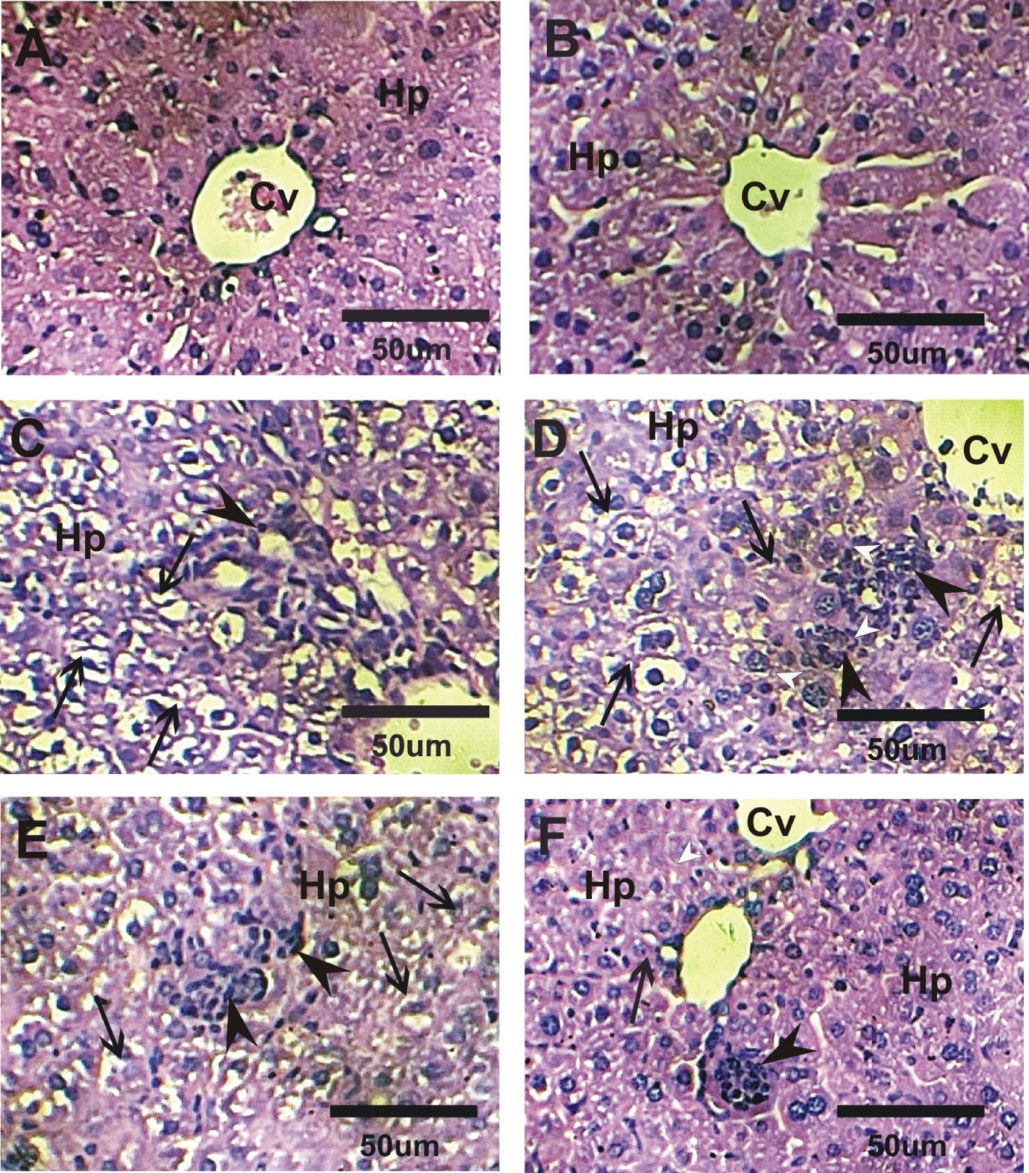
Photomicrographs of liver sections stained with H&E in different groups. **A&B**: Normal radially arranged cords of hepatocytes (Hp) and central vein (Cv) in control and GLONF groups. **C&D**: Liver sections in EST group exhibited marked degeneration with multinucleated giant cells in hepatic cords, marked vaculation (arrows), atrophy, marked inflammatory cells and marked diffuse necrosis (arrow heads). **E**: Liver sections in GLONF + EST group exhibited moderate diffuse necrosis of hepatic tissue (arrow heads), moderate cytoplasmic vacuolization of hepatocytes (arrows) and moderate inflammatory cells. **F**: Liver sections in EST+GLONF exhibited mild inflammatory cells and mild vaculation (arrows).

Liver histology in the control and GLONF groups showed normal architecture, with radially arranged cords of hepatocytes extending from the central vein toward the portal tracts (Fig. 6A and B). In contrast, the EST group displayed severe hepatic damage, including degeneration of hepatocytes, multinucleated giant cells, marked cytoplasmic vacuolation, atrophy, moderate fibrosis, prominent inflammatory infiltration, and extensive necrosis (Fig. 6C and D). In the GLONF + EST group, liver sections showed moderate necrosis, cytoplasmic vacuolation, and inflammation, whereas post-treatment with GLONF (EST+GLONF) resulted in only mild inflammatory cell infiltration and minimal vacuolation, indicating substantial histological recovery (Fig. 6E and F).

### Impact of GLONF in liver PCNA expression

**Fig. 7:**
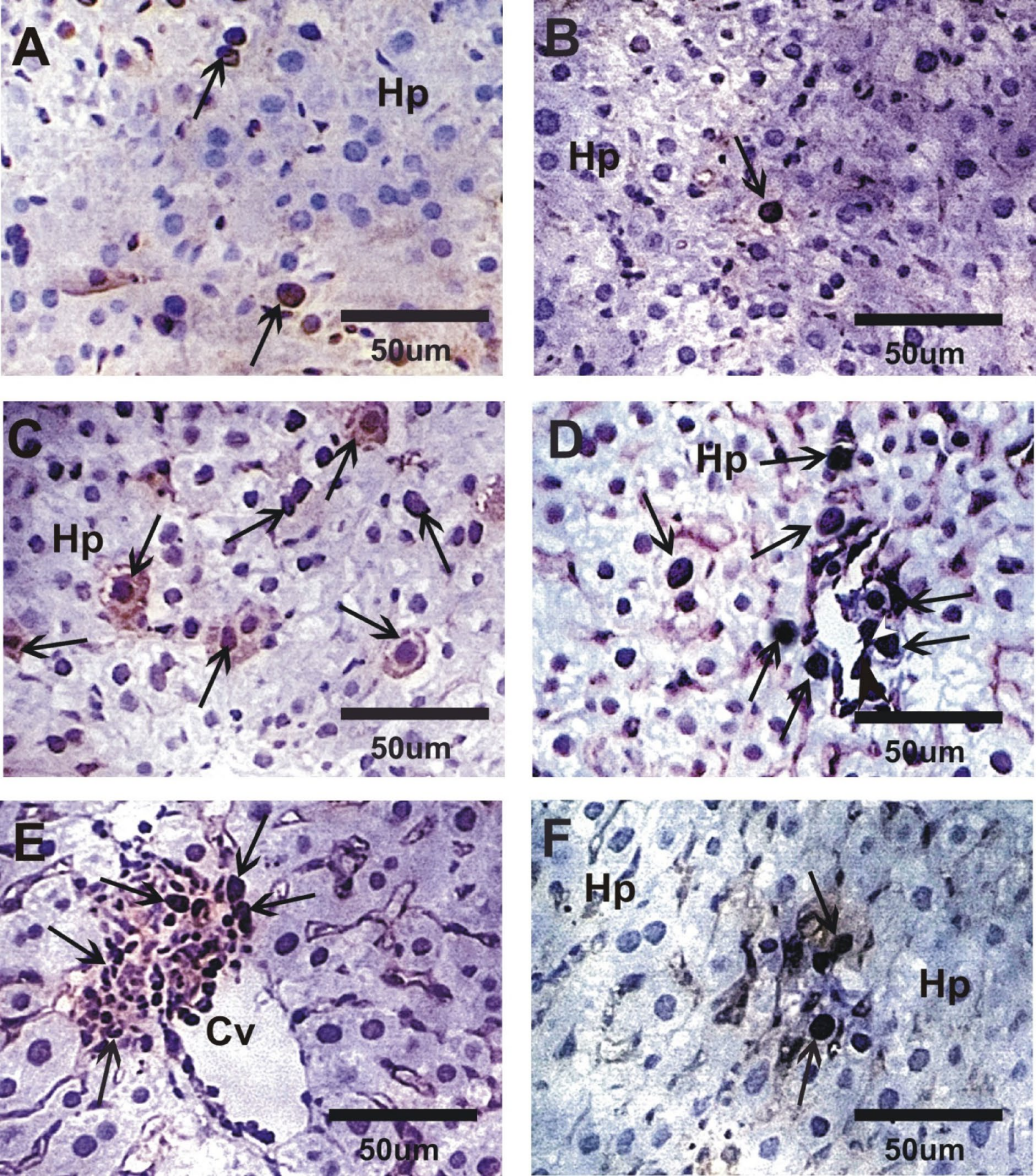
Photomicrographs of liver sections stained with PCNA in different groups. **A&B**: Negative or faint reactions in control and GLONF groups. C&D: Strong reaction for PCNA expressions in EST group. D&E: Moderate reaction was detected in GLONF + EST while mild reaction in EST+GLONF group.

Liver sections from the control and GLONF groups exhibited negative or faint PCNA immunoreactivity (Fig. 7A and B). In contrast, the EST group showed a strong positive reaction for PCNA (Fig. 7C and D). Treatment of EST with GLONF (GLONF + EST or EST+GLONF) markedly reduced PCNA expression, with a moderate reaction observed in the GLONF + EST group and a mild reaction in the EST+GLONF group, indicating a greater recovery following post-treatment (Fig. 7E and F).

### Impact of GLONF in liver P53 expression

**Fig. 8:**
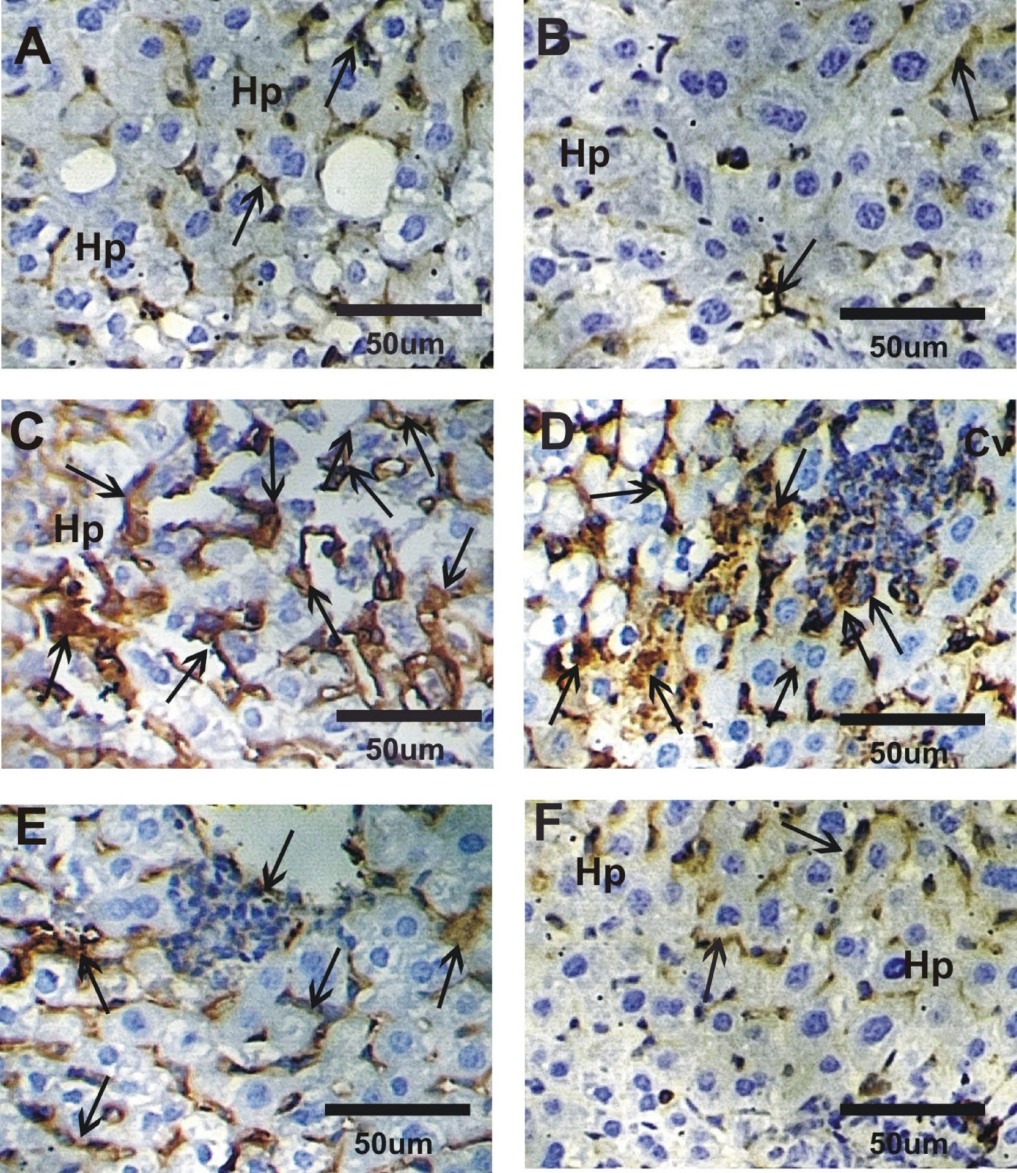
Photomicrographs of liver sections stained with P53 in different groups. **A&B**: Negative or faint reactions in control and GLONF groups. C&D: Strong reaction for P53 expressions in EST group. D&E: Moderate reaction was detected in GLONF + EST while mild reaction in EST+GLONF group.

Liver sections from the control and GLONF groups showed negative or faint positive P53 immunoreactivity (Fig. 8A and B). In contrast, the EST group exhibited strong P53 expression (Fig. 8C and D). Treatment of EST with GLONF (GLONF + EST or EST+GLONF) significantly reduced P53 expression, with a moderate reaction in the GLONF + EST group and a mild to faint reaction in the EST+GLONF group, indicating greater protective effects with post-treatment (Fig. 8E and F).

## Discussion

Cancer is a disease characterized by uncontrolled cell proliferation, resulting in tumor formation and rapid dissemination via the blood and lymphatic systems to distant sites in the body, where they undergo further proliferation of malignant cells leads to tissue destruction and cell death^3^. Ehrlich carcinoma is considered comparable to human cancers because it is undifferentiated, exhibits rapid growth, and shows high sensitivity to chemotherapeutic agents^38^. Several approaches are used for cancer treatment, with chemotherapy remaining one of the most common^13,20^. However, the effectiveness of chemotherapy is often limited by the development of drug resistance in cancer cells and by the cytotoxic effects of chemotherapeutic agents on healthy tissues^37^. Consequently, recent research has focused on the use of natural compounds, including those formulated as nanoparticles, as alternative or complementary anticancer therapies^10,25^..

In the present study, a novel tri-component nano-system based on Sr₀.₅Mn₀.₅La₀.₀₂Fe₁.₉₈O₄ spinel nano-ferrite, chitosan, and glucosodiene was successfully designed as a multifunctional platform for potential biomedical and anticancer applications. To the best of our knowledge, this specific ferrite composition has not been previously synthesized or investigated within the context of nanomedicine, particularly in cancer therapy. The integration of a magnetically active spinel core with a biocompatible polymeric shell and a bioactive therapeutic agent represents a synergistic strategy toward theranostic nanoplatform development^39,40^.

To ensure the reliability and reproducibility of the proposed nano-system, the synthesis of Sr₀.₅Mn₀.₅La₀.₀₂Fe₁.₉₈O₄ spinel nano-ferrite and its subsequent coating with chitosan and glucosodiene were independently repeated in three separate batches under identical experimental conditions. Each batch was subjected to comprehensive physicochemical characterization, including X-ray diffraction (XRD) for phase confirmation, transmission/scanning electron microscopy (TEM/SEM) for morphological analysis, Fourier-transform infrared spectroscopy (FTIR) for surface functional group verification, and vibrating sample magnetometry (VSM) for magnetic property evaluation. The results demonstrated consistent crystallographic phase formation without secondary impurities, comparable particle size distributions, uniform polymer coating, and reproducible magnetic parameters across all batches. Variations in key parameters such as crystallite size, saturation magnetization, and coercivity were minimal and remained within acceptable experimental deviation limits, confirming the robustness of the synthesis protocol and the stability of the tri-component nano-system.

Chitosan serves as a vital biopolymeric interface by providing biocompatibility and stabilization, its characteristics include preventing nanoparticle aggregation, reducing cytotoxicity, improving cellular uptake, and allowing controlled drug release—especially effective in acidic tumor environments^27,28^. Glucosodiene, integrated into this system, contributes antioxidant and anticancer mechanisms while improving solubility and bioavailability. Current research highlights a synergistic nano-platform comprising magnetic functionality (Sr₀.₅Mn₀.₅La₀.₀₂Fe₁.₉₈O₄), biopolymeric functionality (chitosan), and therapeutic functionality (glucosodiene), enabling magnetic targeting, localized hyperthermia, and controlled drug release. This system aims for improved therapeutic efficacy through a multifaceted approach, enhancing cancer treatment outcomes.

Current study demonstrated that EST-bearing mice exhibited a significant increase in serum AST, ALT, and ALP levels, accompanied by a significant decrease in serum albumin and total protein, indicating hepatic injury induced by EST. The observed reduction in albumin and total protein reflects impaired liver biosynthetic function, while the elevated liver enzymes (AST, ALT, ALP) suggest disruption of hepatic metabolism, increased cell membrane permeability, and hepatocyte damage, leading to enzyme release into the bloodstream. These findings are consistent with previous reports by Elgharabawy et al.^25^, Radwan et al.^9^, Askarizadeh et al.^41^, Elkholy et al.^13^ and Abd Eldaim et al.^42^ who reported that; EST-induced elevation of AST, ALT, ALP and depletion in total protein and albumin. Notably, treatment of EST-bearing mice with GLONF effectively mitigated these liver function alterations, indicating its hepatoprotective effect.

The hepatoprotective effect of GLONF may be attributed to its antioxidant and free radical–scavenging properties, which enhance the endogenous antioxidant defense system and reduce ROS-induced cellular injury. Additionally, GLONF may stabilize hepatocyte membranes, thereby preventing enzyme leakage and maintaining hepatic integrity. These findings align with those of Nofal et al.^10^ and Vietrova et al.^44^, who reported that antioxidant-based treatments effectively ameliorate hepatic injury in tumor-bearing models. Overall, the current results suggest that GLONF exerts a significant protective effect against Ehrlich carcinoma–induced hepatic dysfunction. This protective action is likely mediated through its antioxidative mechanisms and its ability to preserve hepatocellular membrane stability. Further studies are recommended to elucidate the precise molecular pathways underlying GLONF’s antitumor and hepatoprotective activities and to evaluate its potential as an adjuvant therapeutic agent in cancer management.

Oxidative stress plays a central role in estrogen-induced liver injury by increasing ROS, leading to lipid peroxidation, antioxidant depletion, and cell damage^45^. In this study, EST elevated MDA levels and reduced GSH, SOD, and CAT. Glucosodiene may act as a free radical scavenger, while the nanoferrite core may provide redox-modulating activity, together enhancing antioxidant defense. These findings align with previous reports by Elsadek et al.⁴⁶, and Abd El-Aziz et al.⁴⁷, who reported that; EST elevate MDA and reducing catalase and SOD activities.

Treatment with GLONF significantly reduced oxidative damage induced by EST, evidenced by decreased MDA levels and improved antioxidant enzyme activities. Both preventive and therapeutic regimens indicated that GLONF enhances hepatic recovery by scavenging reactive oxygen species (ROS) and restoring redox balance. Additionally, glucosodiene contributes to cytoprotection through ROS reduction and affects mitochondrial functions, while chitosan offers some antioxidant properties. The biological activities observed may stem from redox regulation rather than solely magnetic hyperthermia, suggesting that the nano-system could positively influence cellular stress responses and therapeutic effectiveness.

Histological examination further substantiated the biochemical findings. Livers from EST-treated animals exhibited extensive degenerative changes, including hepatocellular vacuolation, inflammatory infiltration, fibrosis, and diffuse necrosis. The observed hepatic injury may be attributed to oxidative stress resulting from excessive production of ROS during tumor progression. ROS can disrupt the structural integrity of hepatocyte membranes, leading to increased permeability and subsequent leakage of intracellular enzymes into the bloodstream^9^. These findings are consistent with previous reports by Abd Eldaim et al^41^., Askarizadeh et al.^43^ and Elkholy et al.^13^ who reported that; EST-bearing mice induced liver injury. In contrast, GLONF administration markedly improved hepatic architecture, with the EST+GLONF group showing only mild inflammatory infiltration and minimal vacuolation. The preservation of normal hepatic structure supports the biochemical evidence of GLONF-mediated liver protection.

Cellular proliferation and apoptosis markers, specifically PCNA and P53, provide insights into liver injury mechanisms. PCNA, a marker of DNA synthesis, indicates hepatocyte proliferation in response to liver damage and is used to evaluate regenerative activity^25^. In contrast, P53, a regulator of cellular stress responses, signals DNA damage and apoptosis when elevated^37^. Thus, analyzing these markers can assess the effectiveness of treatments in mitigating liver injury and stress-induced cellular outcomes.

PCNA expression was significantly increased in the EST group, indicating enhanced hepatocyte proliferation due to injury, consistent with findings by Elkholy et al.^13^ P53 expression was also strongly upregulated, revealing activation of DNA damage-induced apoptotic pathways. In control and GLONF groups, low p53 immunoreactivity suggests normal levels in non-stressed cells, which should not be viewed as pathological. The pronounced P53 expression in the EST group aligns with Nakopoulou et al.^48^, corroborating the link between oxidative stress, aberrant cell proliferation, and apoptotic signaling in hepatic carcinogenesis.

GLONF treatment significantly reduced PCNA and P53 expressions, particularly in the EST+GLONF group, indicating its role in mitigating hepatic injury. This is achieved through oxidative stress reduction, cell cycle limitation, and apoptosis prevention. GLONF’s hepatoprotective effect is linked to its antioxidant properties, stabilization of cellular membranes, and enhancement of liver defense mechanisms against oxidative damage. The suppression of P53 signaling suggests protection against DNA damage and apoptosis. Overall, GLONF attenuates estrogen-induced hepatic injury, with greater effects noted post-treatment, although further research is needed to understand the molecular pathways and long-term safety.

## Conclusion

The Sr₀.₅Mn₀.₅La₀.₀₂Fe₁.₉₈O₄ spinel nano-ferrite–chitosan–glucosodiene nano-system (GLONF) is a groundbreaking multifunctional nanoplatform with magnetic, polymeric, and therapeutic properties aimed at cancer therapy. GLONF demonstrates hepatoprotective effects by reducing oxidative stress and improving drug delivery and tumor targeting, suggesting its potential for clinical treatments pending further safety evaluations.

## Data Availability

The datasets used and/or analyzed during the current study are available from the corresponding author on reasonable request.
